# A Usage Aware Dynamic Spectrum Access Scheme for Interweave Cognitive Radio Network by Exploiting Deep Reinforcement Learning

**DOI:** 10.3390/s22186949

**Published:** 2022-09-14

**Authors:** Xiaoyan Wang, Yuto Teraki, Masahiro Umehira, Hao Zhou, Yusheng Ji

**Affiliations:** 1 Graduate School of Science and Engineering, Ibaraki University, Mito 310-8512, Japan; 2 IVIS Cooperation, Tokyo 113-0033, Japan; 3 Faculty of Science and Technology, Nanzan University, Nagoya 466-0824, Japan; 4 School of Computer Science, University of Science and Technology of China, Hefei 230052, China; 5 Information Systems Architecture Research Division, National Institute of Informatics, Tokyo 101-8430, Japan

**Keywords:** dynamic spectrum access, interweave cognitive radio, deep reinforcement learning, channel usage aware, spectral utilization efficiency, interference violation

## Abstract

Future-generation wireless networks should accommodate surging growth in mobile data traffic and support an increasingly high density of wireless devices. Consequently, as the demand for spectrum continues to skyrocket, a severe shortage of spectrum resources for wireless networks will reach unprecedented levels of challenge in the near future. To deal with the emerging spectrum-shortage problem, dynamic spectrum access techniques have attracted a great deal of attention in both academia and industry. By exploiting the cognitive radio techniques, secondary users (SUs) are capable of accessing the underutilized spectrum holes of the primary users (PUs) to increase the whole system’s spectral efficiency with minimum interference violations. In this paper, we mathematically formulate the spectrum access problem for interweave cognitive radio networks, and propose a usage-aware deep reinforcement learning based scheme to solve it, which exploits the historical channel usage data to learn the time correlation and channel correlation of the PU channels. We evaluated the performance of the proposed approach by extensive simulations in both uncorrelated and correlated PU channel usage cases. The evaluation results validate the superiority of the proposed scheme in terms of channel access success probability and SU-PU interference probability, by comparing it with ideal results and existing methods.

## 1. Introduction

In the past decade, mobile data traffic has grown tremendously due to the increase of wireless communication terminals and spectrum-hungry applications. The monthly global data traffic reached 77 exabytes in 2022, i.e., a seven-fold increase over 2017, which is predicted to reach 131 exabytes per month by 2024. This blossoming traffic demand is driving the need for either improved spectrum efficiency in traditional sub-6 GHz frequency band or the utilization of additional spectrum in millimeter wave (mmWave) frequency bands. The wireless communication in mmWave band has a huge potential, however, its high blockage and scattering losses characteristics limit the mmWave band use cases. On the other hand, the spectrum resource below 6 GHz is comparatively easy to use for mobile communication systems. However, the frequency bands below 6 GHz are already almost fully allocated to various existing wireless systems in a static and exclusive way, e.g., TV broadcasting, video camera services, radar systems, fixed satellite systems, etc. In the current spectrum allocation paradigm, some primary systems have extremely low spectral utilization efficiency, since they have a large amount of unutilized or underutilized spectrum resources in both time and space domains [1]. To this end, dynamic spectrum access (DSA) which is empowered by interweave cognitive radio techniques, has been widely investigated [2,3,4,5], in which the secondary users (SUs) are allowed to access the abundant spectrum holes i.e., whitespaces, in the licensed spectrum bands that belong to the primary users (PUs).

Two kinds of DSA approaches have been extensively studied recently, i.e., database driven spectrum access approaches and opportunistic spectrum access approaches. In the database driven spectrum access approaches [6,7,8,9,10], the SU queries a spectrum database about the spectrum availability information before the channel access. The spectrum database could be either constructed by propagation models [6] or crowdsourced spectrum-sensing measurements [7,8,9,10]. The main concern in this kind of approach is the database’s accuracy and high maintaining/updating cost. In opportunistic spectrum access approaches [11,12,13,14,15,16,17,18], the SU senses or predicts the spectrum holes of PUs, and accesses them dynamically. Ideally, the PUs are oblivious of the presence of SUs, if the SUs do not cause any interference. This approach is cost-efficient but may suffer from severe interference if the sensing or predicting results are not accurate. The key issue for opportunistic spectrum access is to predict the PUs’ channel usage status, and thus let SU access a channel that most likely to be idle to minimize the interference ratio and maximize the system’s total spectral utilization ratio. To this end, neural network model based primary user activity prediction methods were proposed in [13,14,15], with the objective of reducing both the spectrum underutilization and interference violations. In [16], by assuming that the PUs’ channel occupancy pattern obeys an exponential ON-OFF time distribution, a predictive channel selection algorithm was proposed and implemented in a wireless test-bed. Without the assumption of channel usage patterns, model-free spectrum access methods have been extensively investigated by utilizing the learning algorithms, and the details of them will be introduced in Section 2. In our previous work [19], we investigated the dynamic channel access problem in a specific uncorrelated three-PUs scenario, and provided some preliminary results to validate the practicability of the proposed deep reinforcement learning based method. However, the method proposed in [19] is dedicated to a very specific case without formal formulation, and is hard to be extended to general channel access problem.

Based on the idea and preliminary results in [19], in this work, we consider a general dynamic channel access problem in a multiple-PU single-SU interweave cognitive radio network, by taking consideration both correlated and uncorrelated PU channel usage patterns. We mathematically formulate this problem to an optimization problem with the goal of maximizing the spectrum access success ratio and minimizing the interference violation ratio. We propose a novel usage-aware spectrum access scheme by exploiting deep reinforcement learning technique [20], in which the SU acts as an agent who could learn the optimal channel access policy by interacting with the wireless environment in a trial-and-error manner. Specifically, the proposed scheme exploits a deeper historical channel usage data of PUs by a compressed status representation method, and uses a usage-status-aware reward function to solve the reward sparsity problem. Moreover, to reduce the interference probability when the whitespace of PU is very limited, an additional no access option is provided to further reduce the interference ratio. We perform extensive simulations to evaluate the performance of the proposed scheme by using a new evaluation metric which is defined as the difference between the channel access success probability and the SU-PU interference probability. The evaluation results demonstrate that our proposed scheme constantly keeps a small gap between the ideal results and outperforms the existing methods significantly under different PU channel usage patterns.

The rest of the paper is organized as follows. Section 2 introduces the related works. Section 3 describes the system model and preliminaries of reinforcement learning and deep reinforcement learning techniques. Section 4 presents the proposed scheme in detail. Finally Section 5 provides the evaluation results, and Section 6 draws the conclusions.

## 2. Related Work

The underutilization of sub-6GHz frequency bands caused by current spectrum allocation policy has stimulated a flurry of research activities in opportunistic spectrum access. Besides the conventional dynamic programming [21] and game theory [22] based channel access approaches, model-free learning-based approaches are widely addressed. These researches intend to keep track of PUs’ channel usage status, and let SU either sense the most likely idle channel to avoid interference or access the most likely channel in a best-effort way with acceptable interference ratio. Specifically, reinforcement learning based opportunistic spectrum access methods have been employed recently, which formulates the channel access problem as a Markov Decision Process (MDP). An optimal policy is derived to maximize the number of time slots with successfully secondarily used while constraining the interference caused to the PUs. In [23], the channel access problem was formulated as a multi-arm restless bandit process by assuming the system transition is known a priori, and a myopic spectrum access policy was proposed, which designs a sensing policy for channel selection to maximize the average reward. In [24], a restless Multi-armed bandit (MAB) based approach [24] was investigated for homogeneous channel scenarios without the requirements of system transition statistics. In [25], the spectrum sensing order problem in the scenario with idle spectrum across multiple network service providers was investigated, in which a discounted Thompson sampling method was proposed to address the formulated optimization task. In [26,27,28,29], with the assumption that the observable full system states, reinforcement learning based dynamic spectrum access approaches were proposed. In [30], a deep reinforcement learning-based dynamic multi-channel access method was firstly proposed, which takes into consideration the partial observability. In [31], a deep actor-critic reinforcement learning method was proposed for spectrum sensing problem for both single user case and multiple users case. Furthermore, a deep reinforcement learning based distributed dynamic spectrum access scheme for multiple SUs was investigated in [32], which uses a local observation indicating whether its packet was successfully delivered or not as a reward. However, this work assumed that the channel utilizations for PUs are invariant. A deep recurrent Q-network-based dynamic spectrum access method for a scenario with multiple independent channels and multiple heterogeneous PUs was proposed in [33]. Aside from the aforementioned methods that focused on fixed time slot channel sensing, spectrum sensing with adaptive time slot structure have also been studied. In [34], the authors deduced the structure of optimal sensing interval policy for channels with hyper-exponential distribution OFF times through Markov decision process, and used dynamic programming framework to derive sub-optimal sensing interval policies. In [35], the authors addressed the problems of which channel to sense and how often to sense. Specifically, a reinforcement learning based channel selection method and a Bayesian skip sensing duration method were proposed. In [36], the authors considered the tradeoff between sensing and transmission, and proposed a deep reinforcement learning based spectrum sensing strategy with the goal of maximizing the expected achievable throughput of SU. In [37], a reservoir computing-based distributed spectrum access approach was proposed, which takes into consideration the spectrum sensing errors.

## 3. System Model and Preliminaries

### 3.1. System Model

In this paper, we consider a conventional dynamic channel access model for the interweave cognitive radio network, where *N* PUs use *N* respective channels and a single SU tries to opportunistically access the whitespace of PUs for secondary use in a slot-by-slot manner. The PU channel usages could be either correlated or uncorrelated. In each time slot, the usage status of PU is represented by “−1” when it is busy and “1” when it is idle. The PU usage patterns can be characterized by two metrics, duty cycle (DC) and complexity [38]. DC indicates the activity level of PU, which is defined as the time ratio of its presence. Therefore, a high DC means less whitespace is available for secondary use. Complexity is an index showing the degree of irregularity in the channel usage by measuring the rate of production of new patterns. The complexity could be measured by the entropy rate given by Equation (1), which is defined as the expected value of the amount of information that increases when one random variable is added to the random variable sequence.

(1) $$h=-\sum_{ij}\delta_{i}p_{ij}\log p_{ij}.$$

In the context of this paper, $p_{ij}$ denotes the channel status transition rate, which includes $p_{00}$, $p_{01}$, $p_{10}$, $p_{11}$. $\delta_{1}$ represents the DC of PU’s channel usage, and $\delta_{0}=1-\delta_{1}$. The usage pattern of PU with large entropy rate indicates that it has high complexity and thus is hard to predict.

The SU tries to predict all *N* channels usage status on the next time slot, and either access a channel that is most likely to be idle or retrain from channel accessing. If the channel that SU accessed is idle, the spectrum access succeeds and there has no interference between SU and PU. Otherwise, the spectrum access fails and SU-PU interference occurs. In this case, the SU must refrain from the channel accessing. Obviously, accurate channel usage prediction for the next time slot is the key to maximize the spectral utilization ratio and minimize the interference probability.

### 3.2. Preliminaries

#### 3.2.1. Reinforcement Learning

Q-Learning [39] is a representative reinforcement learning algorithm that learns the optimal policy in an interactive environment by trial and error. By assuming discrete time, in time slot *k*, the agent observes the *state*$s_{k}$ of the environment, and takes an *action*$a_{k}$ based on a policy π. Upon the action being taken, the state moves from $s_{k}$ to $s_{k+1}$, and the agent obtains a *reward/cost*$r_{k}$ that indicates the benefit/loss by taking $a_{k}$ at $s_{k}$. The optimal action policy $\pi^{*}$ is computed by maximizing/minimizing the expectation of the future cumulative discounted reward/cost. In Q-learning, a Q-function is defined to represent the expected future cumulative discounted reward for action $a_{k}$ under state $s_{k}$. The values of the Q-function, i.e., Q-value, are stored in a Q-table, whose size is the number of states times the number of actions. The Q-value in time slot *k* is updated by Equation (2).

(2) $$Q_{\left(s_{k},a_{k}\right)}^{\prime }=Q_{\left(s_{k},a_{k}\right)}+\alpha \left(r_{k}+\gamma \min_{a_{k+1}}Q_{\left(s_{k+1},a_{k+1}\right)}-Q_{\left(s_{k},a_{k}\right)}\right),$$

where $Q_{\left(s_{k},a_{k}\right)}^{\prime }$ is the Q-value after update, $Q_{\left(s_{k},a_{k}\right)}$ is the current Q-value, α is the learning rate which is in range 0≤α≤1, $r_{k}$ is the cost, γ is the discount factor which is in range 0≤γ≤1, and $\min_{a_{k+1}}Q_{(s_{k+1},a_{k+1)}}$ represents the minimum Q-value for the actions that the agent can select at next time slot k+1 under the new state $s_{k+1}$. Notice that in Equation (2), minimum Q-value is used, since $r_{k}$ is a cost instead of a reward, i.e., smaller Q-value is more desirable.

#### 3.2.2. Deep Reinforcement Learning

When the spaces of state $s_{k}$ and action $a_{k}$ increase, the Q-table based classic Q-learning method may fail due to the so-called the curse of dimensionality problem, i.e., many state-action pairs are rarely visited and the storage of the table becomes impractical. To solve this problem, Deep Q-Network (DQN) [20] has been proposed, which approximates the Q-table by a neural network. By introducing a weight $\theta^{k}$ of the DQN, the task of finding the best Q-function is transformed to search the best weight $\theta^{k}$. Therefore, the update of the Q-value is represented by Equation (3).

(3) $$\begin{array}{cc} \; & Q_{\left(s_{k},a_{k},\theta^{k}\right)}^{\prime }=Q_{\left(s_{k},a_{k},\theta^{k}\right)}+\\ & \alpha \left(\left(1-\gamma \right)r_{k}+\gamma \min_{a_{k+1}}Q_{\left(s_{k+1},a_{k+1},\tilde{\theta^{k}}\right)}-Q_{\left(s_{k},a_{k},\theta^{k+1}\right)}\right). \end{array}$$

A replay memory is used to store the latest *U* state-action-cost tuples, such as $\varphi =\{m^{\left(k-U+1\right)},\cdots ,m^{k}\}$, where $m^{k}=\left\{s_{k},a_{k},r_{k},s_{\left(k+1\right)}\right\}$. A mini-batch $\tilde{\varphi }\in \varphi$ is sampled from the replay memory instead of the most recent experience to calculate the loss function, which is defined as the difference between a target Q value and the current Q value. The weight $\theta^{k}$ of the neural network is updated by the gradient descent method. To make the training more stable, a target Q-network is used to back-propagate through and train the main Q-network. The loss function and the gradient are given by Equations (4) and (5), respectively. By utilizing DQN, the agent could learn the optimal Q-value for a state-action pair in a semi-online fashion.

(4) $$\begin{array}{cc} \; & L\left(\theta^{k+1}\right)=E_{\left\{s_{k},a_{k},r_{k},s_{k+1}\right\}\in \tilde{\varphi }}[(\left(1-\gamma \right)r_{k}\\ \; & +\gamma Q\left(s_{k+1},\underset{a_{k+1}}{arg min}Q\left(s_{k+1},a_{k+1},\tilde{\theta^{k}}\right),\theta^{k}\right)-Q\left(s_{k},q_{k},\theta^{k+1}\right){)}^{2}], \end{array}$$

(5) $$\begin{array}{cc} \; & \nabla_{\theta^{k+1}}L\left(\theta^{k+1}\right)=E_{\left\{s_{k},a_{k},r_{k},s_{k+1}\right\}\in \tilde{\varphi }}[(\left(1-\gamma \right)r_{k}\\ \; & +\gamma Q\left(s_{k+1},\underset{a_{k+1}}{arg min}Q\left(s_{k+1},a_{k+1},\tilde{\theta^{k}}\right),\theta^{k}\right)-Q\left(s_{k},a_{k},\theta^{k+1}\right))\\ \; & \nabla_{\theta^{k+1}}Q\left(s_{k},a_{k},\theta^{k+1}\right)]. \end{array}$$

## 4. Proposed Deep Reinforcement Learning Based Usage Aware Spectrum Access Scheme

In this section, we firstly mathematically formulate the dynamic spectrum access problem in a interweave cognitive radio network with multiple-PU and single-SU. Then, we propose a deep reinforcement learning based usage aware spectrum access scheme to let the SU predict the channel usage at next time slot and access the most likely idle channel. The goal of the proposal is to maximize the system spectral utilization efficiency and minimize the SU-PU interference violations.

### 4.1. Problem Formulation

In this paper, we consider a typical spectrum access model with *K* PUs and single SU. The time is discretized into time slots, and the idle or busy status of PU does not change during one time slot. The status of total *K* PUs’ channels at time slot *t* is denoted by $s^{t}=\left[s_{1}^{t},s_{2}^{t},\cdots ,s_{K}^{t}\right]$, where $s_{k}^{t}=1$ if the *k*-th PU’s channel is idle at time slot *t*, and $s_{k}^{t}=-1$ if it is busy. At time slot *t*, SU determines whether accessing the channel or not and which channel to access if so. A channel access indicator at time slot *t* is represented by $a^{t}=\left[a_{1}^{t},a_{2}^{t},\cdots ,a_{K}^{t}\right]$, where $a_{k}^{t}=1$ if SU accesses the *k*-th channel at next time slot and $a_{k}^{t}=0$ otherwise. Notice that $\sum_{k=1}^{K}a_{k}^{t}\leq 1$, which indicates that SU can at most access one channel at a time. Accessing an idle channel leads to a success spectrum reuse, and accessing a busy channel results in interference and thus the SU must refrain from accessing.

The SU aims at finding a series of optimal channel access policy in total time slots *T*, so as to maximize the number of time slots that have been successfully accessed and minimize the number of time slots in which the interference occurred. The problem could be formulated as follows.

(6) $$\begin{array}{cc} \; & \;\;\max_{a^{1},a^{2},\cdots ,a^{T}}\sum_{t=1}^{T}{\left(s^{t}\right)}^{T}a^{t},\\ \; & s.t.\;\sum_{k=1}^{K}a_{k}^{t}\leq 1,\;a_{k}^{t}\in \left\{0,1\right\} \end{array}$$

where the constraint indicates that the SU could at most access one channel for each time slot *t*. For each time slot *t*, the value of the objective function in Equation (6) has three possible cases, i.e., 1 when SU accesses an idle channel, 0 when SU refrains from channel access, and −1 when SU accesses a busy channel.

### 4.2. Existing Q-Learning and DQN Based Spectrum Access Methods

**Figure 1:**
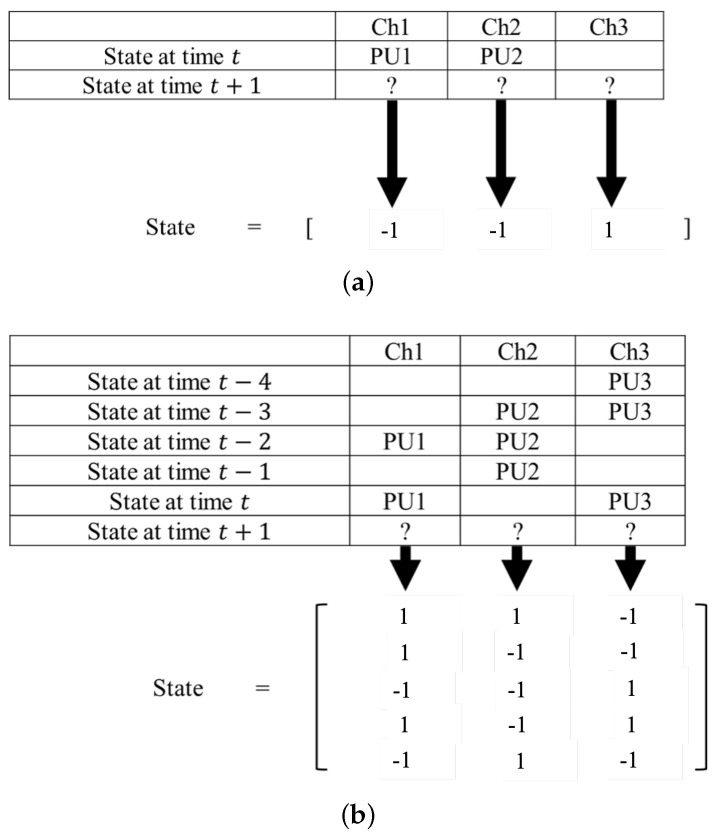
An illustration of the state definitions for the existing methods. (**a**) Q-learning based spectrum access method. (**b**) DQN based spectrum access method.

Before presenting the proposed scheme, we briefly introduce the basic idea of the existing Q-learning and DQN based spectrum access methods. The Q-Learning based spectrum access method uses the channel usage status at current time slot *t* of different PUs as the states for learning. An illustrative example with 3 PUs is shown in Figure 1a. If at time slot *t*, the status of channels #1,#2,#3 are busy, busy and idle, respectively, the states will be recorded by vector [−1,−1,1]. The action will be the index of the channel that SU intends to access at next time slot t+1. For instance, $a^{t}=\left[0,0,1\right]$ indicates that SU will access channel #3 at next time slot. If the channel that SU intends to access at time slot t+1 is idle, the spectrum secondary use succeeds and the system’s total spectral utilization ratio improves. Otherwise, if the channel that SU accesses at time slot t+1 is busy, the channel access fails since the interference occurs. The cost value is defined as 0 if the channel access succeeds, or 1 otherwise.

In the DQN based spectrum access method, the historical channel usage status is used as the states for learning. The original Q-table based learning method cannot exploit the historical data, since it leads to a tremendous increase of the state-action space. For instance, in the same scenario that the number of PUs is 3, if the past channel usage status back to time slot t−4 is applied, the number of states will increase rapidly from 2×2×2=8 to ${\left(2\times 2\times 2\right)}^{5}=32,768$. Hopefully, the DQN algorithm approximates the Q-table by neural network, and thus is capable of dealing with complicated scenario with huge state-action space.

We briefly explain the DQN based spectrum access method by using the same PU = 3 example. Instead of only using the current time slot *t*’s status, the historical channel usage status back to time slot t−4 is applied. For the example channel usage history that given in Figure 1b, the state matrix can be represented as [1,1,−1;1,−1,−1;−1,−1,1;1,−1,1;−1,1,−1]. Here, the first row records 1,1,−1 represent the usage status for channels #1,#2,#3 at time slot t−4, the second row records 1,−1,−1 represent the usage status for channels #1,#2,#3 at time slot t−3, and so on. Action set and cost value are similarly defined as that in the Q-learning based spectrum access method described above. Specifically, the action set has three possible actions since there are three channels and, the cost value is set to 0 when the channel access succeeds, and the cost is set to 1 when the channel access fails.

### 4.3. Proposed Usage Aware Spectrum Access Scheme

In this subsection, we present a novel deep reinforcement learning based usage aware spectrum access scheme. To improve the training performance, a double Q-network architecture is applied, in which one is used to determine the action and another is used to evaluate the action. Furthermore, a replay memory is utilized, and the agent randomly gathers a mini-batch from the replay memory, and uses it to update the neural network to approximate the Q-value function. The proposed scheme improves the previous existing methods in three aspects: compressed states representation, additional action and status aware cost functions.

#### 4.3.1. Compressed States Representation

**Figure 2:**
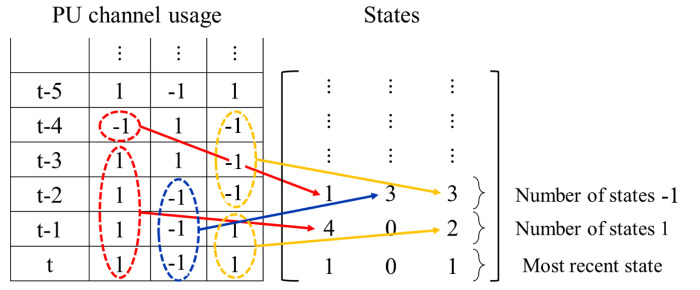
An illustration of the proposed compressed states representation.

In the existing Q-learning and DQN based spectrum access methods, the state vector and matrix directly record the current and historical PU channel usage status. Specifically, 1 or −1 represents a specific channel for a specific time slot is idle or busy, respectively. In this paper, in order to exploit a deeper historical PU usage status with a same state matrix size, we propose a compressed state representation method, in which the number of continuous channel status and the current channel status are recorded. To facilitate better understanding, Figure 2 shows an example of how the proposed compressed states represents the current and historical channel usages in a three-PU-channels scenario. The compressed state is a matrix, in which each column records the channel usage information of a specific channel. The last row, i.e., “101” in the example, represents the latest channel usage status for three channels, i.e., $s_{1}^{t},s_{2}^{t},s_{3}^{t}$. Notice that “−1” which denotes busy is changed to “0” in the state matrix to let all the elements in the state matrix non-negative, and the remaining rows record the number of consecutive “−1” (busy) and “1” (idle) alternately for different channels. For instance, the usage status for channel #1 at time slots t−3,t−2,t−1,t, i.e., $s_{1}^{t-3},s_{1}^{t-2},s_{1}^{t-1},s_{1}^{t}$, are recorded as 4 in the compressed state matrix. The total size of the compressed states matrix is fixed, therefore, once the channel status is switched twice, the oldest information on the first and second rows is deleted, and the records following behind will be shifted forward. By using the proposed compressed states representation, more information regarding the historical channel usages could be recorded by the same size of state matrix.

#### 4.3.2. Additional Action

In the existing methods, the SU must take an action to choose and access a channel at the next time slot. However, in the scenario that the DCs of all the PU channel usages are high, the available whitespace is limited and thus interference between SU and PU is extremely hard to avoid. To this end, we add an additional “no access” action to the action set. Specifically, $a_{k}^{t}=0$ indicates that the SU refrains from accessing channel *k* at the next time slot, therefore $|a^{t}|=0$ denotes that the SU does not access any channel at next time slot.

#### 4.3.3. Status Aware Cost Function

In the existing Q-learning and DQN based spectrum access methods, the cost is set to either “0” or “1” depending on the channel access is successful or not. This cost setting has two problems. The first one is that the sparse cost value setting has a negative impact on the learning process and may result in an unstable learning result. The second one is that the cost setting has not taken into consideration the no access case. To this end, in the proposed scheme, the cost functions are delicately designed, which has several discrete values based on the PU channel usage status and SU’s actions. The cost value is assigned based on the state-action pair’s “inappropriate level”. The basic idea is that, the most appropriate one is assigned to a cost value equals to 0, and the most inappropriate one is assigned to a cost value equals to 1. Specifically, the cost value calculation for all the possible state-action pairs is defined in Equation (7), which is related to the PU channel usage status and SU’s corresponding actions. For instance, choosing no access action when all the channels are idle is more inappropriate compared with that when only one channel is idle.

(7) $$C^{t}=\left\{\begin{array}{cc} \frac{N_{\delta }}{N}, & |a^{t}|=0\\ \frac{N_{\delta }}{2(N-1)}+0.5, & a_{k}^{t}=1\;\&\;s_{k}^{t}=-1\\ 0, & a_{k}^{t}=1\;\&\;s_{k}^{t}=1 \end{array}\right.$$

**Figure 3:**
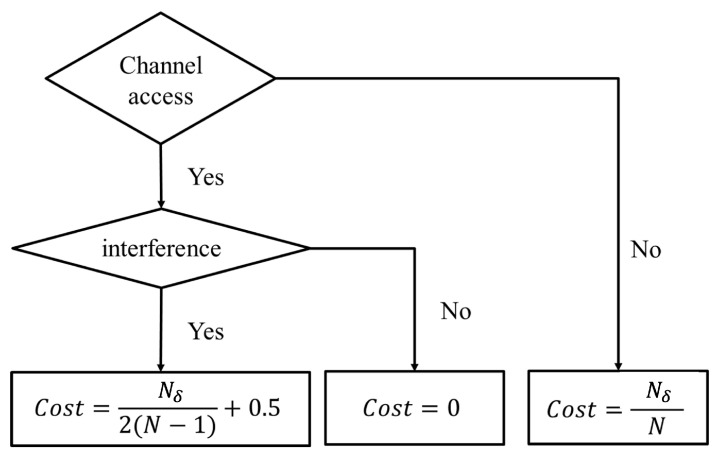
An flowchart of the proposed status aware cost function.

Here, $N_{\delta }$ and *N* denote the numbers of idle channels and total channels, respectively. $N_{\delta }$ could be easily derived by measuring the total received power. Notice that $N_{\delta }$ is required only when the SU does not access the channel or it has to refrain from accessing due to interference occurring. Specifically, if SU chooses the no access action, the cost value will increase in proportion to the number of idle channels. If SU chooses to access the channel *k*, and unfortunately interference occurs, the cost will proportionally increase with the number of idle channels in the range $[0.5,1]$. Finally, if SU chooses to access the channel *k*, and channel *k* is idle, then the cost will be 0. A flowchart to illustrate the proposed status aware cost function design is given by Figure 3.

## 5. Simulation Results

### 5.1. Simulation Settings

**Figure 4:**
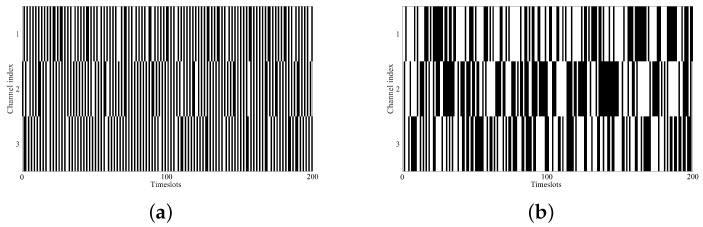
A channel usage example for 3 PU channels (DC = 0.5). (**a**) Pattern $\eta_{1}$. (**b**) Pattern $\eta_{9}$.

In the simulation, we considered a typical interweave cognitive radio network with multiple PUs and single SU. Regarding the PU channels, both the traditional correlated channels and extremely challenging uncorrelated channels are considered. Various DCs and complexities for the PU channel usages are adopted. The DC varies in range [0.1,0.9]. For each DC, nine patterns of data with different complexities are used, and the complexity is measured by entropy rate that given by Equation (1). The entropy rates for each DC are shown in Table 1. From pattern $\eta_{1}$ to pattern $\eta_{9}$, the complexities gradually increase. An example of the PU channel usages for DC = 0.5 with pattern $\eta_{1}$ and pattern $\eta_{9}$ are illustrated in Figure 4. It is obvious that the channel usage pattern $\eta_{1}$ with low complexity is much easier to predict compared with pattern $\eta_{9}$.

To validate the performance of the proposed scheme, we compare it with the ideal results, random channel access method, Q-learning based method [26], and DQN based method [30]. The ideal result is obtained by assuming that the SU has the perfect knowledge of all PUs’ channel usage status at all future time slots, which provides an upperbound for the performance evaluation. The random channel access method is a baseline method, in which the SU randomly picks a channel and accesses it at the next time slot. The Q-learning and DQN learning based methods are introduced in Section 4.2. The proposed scheme’s major parameters are summarized in Table 2. We use Matlab [40] to generate the channel usage time series with different DCs and complexities, and Python [41] to derive the ideal result and realize the proposed scheme, the DQN-based method, the Q-learning based method and the random channel access method.

In this paper, we aim at maximizing the SU’s channel access success probability and minimizing the SU-PU interference probability simultaneously. Therefore, we define a new evaluation metric, i.e., $P_{s}-P_{i}$, which is the channel access success probability $P_{s}$, minus the SU-PU interference probability $P_{i}$. The range of the evaluation metric $P_{s}-P_{i}$ will be from −1 to 1. In real applications, PU will inform the interfering SU to refrain from transmitting once the interference is occurred.

### 5.2. Evaluation Results for Correlated Channel Usages

First, we validate the proposed method in correlated channel usages cases. In this setting, 10 PU channels are evenly divided into two groups, i.e., $G_{1}$ with five channels and $G_{2}$ with five channels. In each group, the channels’ idle and busy states changes with the same pattern. To keep the total DC as 0.5, we consider three kinds of combination for $G_{1}$ and $G_{2}$, specifically DC($G_{1}$) = 0.1 and DC($G_{2}$) = 0.9; DC($G_{1}$) = 0.3 and DC($G_{2}$) = 0.7; DC($G_{1}$) = 0.5 and DC($G_{2}$) = 0.5.

**Figure 5:**
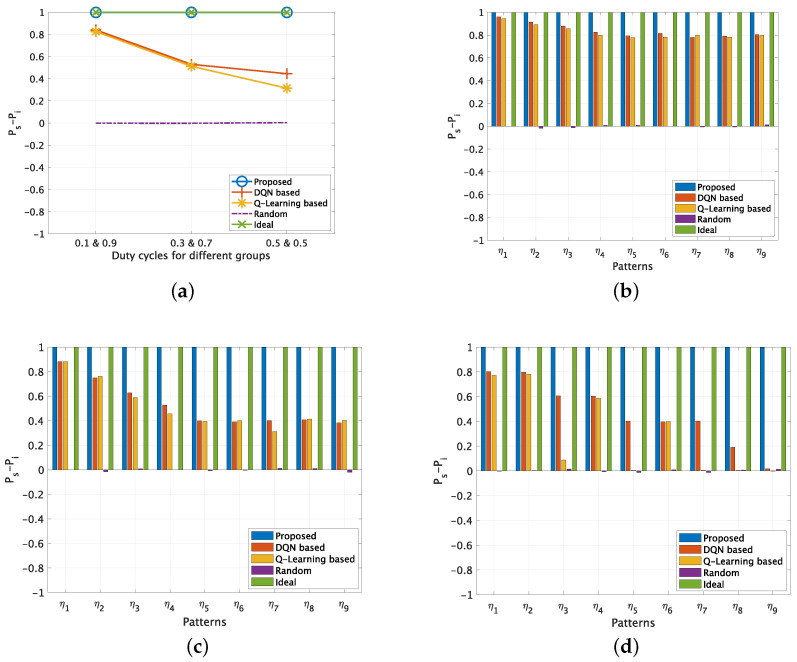
$P_{s}-P_{i}$ for correlated PU channels (No. of PU = 10). (**a**) Different DCs for groups $G_{1}$ and $G_{2}$. (**b**) Different patterns when DC($G_{1}$) = 0.1 and DC($G_{2}$) = 0.9. (**c**) Different patterns when DC($G_{1}$) = 0.3 and DC($G_{2}$) = 0.7. (**d**) Different patterns when DC($G_{1}$) = 0.5 and DC($G_{2}$) = 0.5.

First, Figure 5a illustrates the results of $P_{s}-P_{i}$ for different DC combinations. It is obvious that the ideal results are always 1, since the channel access success probability is 100% and the SU-PU interference probability is 0%. The random channel access method could only achieve $P_{s}-P_{i}=0$, since its channel access success probability and SU-PU interference probability are both 50%. For the proposed method, it could achieve almost the same performance as the ideal results for all three duty cycle combinations, which indicates that both the time correlation and channel correlation of the PU channels are well learned. On the other hand, the performance of two existing methods, i.e., the Q-learning based method and the DQN-based method, varies from 0.31 to 0.82 at different duty cycle combinations, and the DQN-based methods performs slightly better than Q-learning based method.

Next, Figure 5b–d shows the results of $P_{s}-P_{i}$ at different DC combinations with varying complexities. Firstly as expected, the performance of the ideal results and random channel access method keeps constant regardless of the complexities. Moreover, it is clear that the performance of the proposed method also does not change with PU channel usage complexities, which achieves almost similar results with ideal results at all complexities. However, it is obvious that the two existing methods’ performance degrades as the PU channel usage complexity increases, especially for the DC($G_{1}$) = 0.5 and DC($G_{2}$) = 0.5 case, and the DQN-based method achieves more stable performance compared with Q-leaning based method. We can observe that the Q-learning based method fails to predict the channel state completely at some scenarios, e.g., $\eta_{3}$, $\eta_{5}$, $\eta_{7}$ and $\eta_{8}$ when DC($G_{1}$) = 0.5 and DC($G_{2}$) = 0.5.

### 5.3. Evaluation Results for Uncorrelated Channel Usages

**Figure 6:**
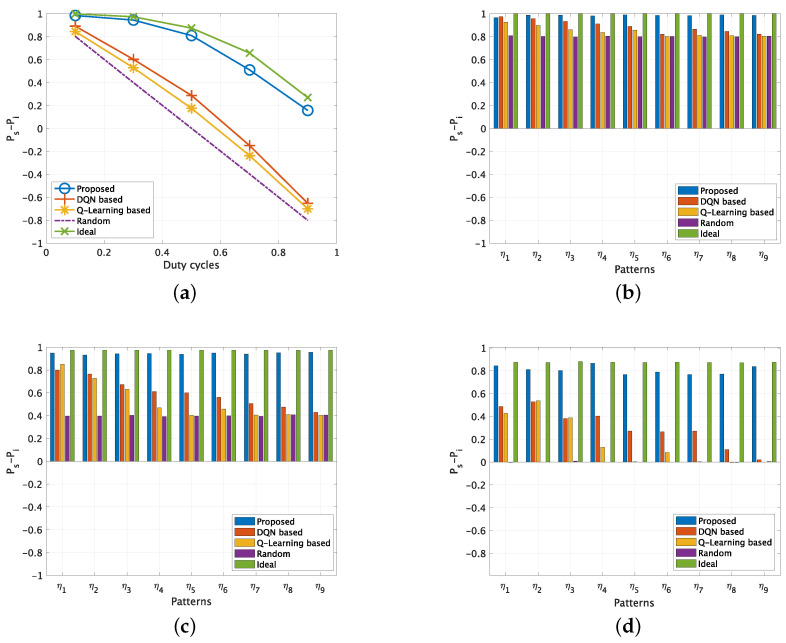

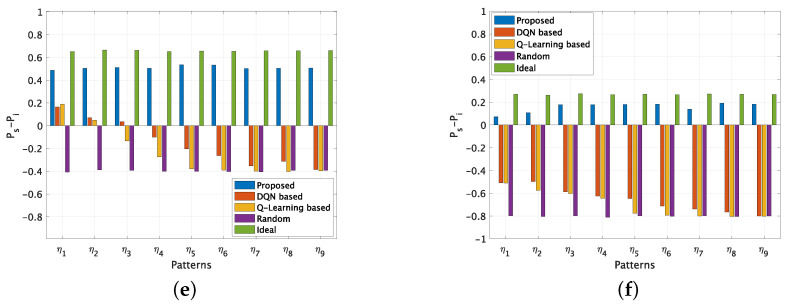
$P_{s}-P_{i}$ for uncorrelated PU channels (No. of PU = 3). (**a**) Varying DCs. (**b**) Different patterns when DC = 0.1. (**c**) Different patterns when DC = 0.3. (**d**) [Different patterns when DC = 0.5. (**e**) Different patterns when DC = 0.7. (**f**) Different patterns when DC = 0.9.

Next, we compare the performance of different methods in an uncorrelated channel usage scenario. First, we show the performance in terms of $P_{s}-P_{i}$ in a 3 PU channel scenario in Figure 6. Figure 6a shows the results of $P_{s}-P_{i}$ varying with DC from 0.1 to 0.9. As expected, when the DC of the PUs increases, it becomes more difficult for the SU to access the channel successfully without interference violation for all the channel access methods. Even for the ideal results, the $P_{s}-P_{i}$ decreases from 1 to 0.26 when the DC increases from 0.1 to 0.9. For the random channel access method, the performance linearly degrades from 0.8 to −0.8 when the DC increases from 0.1 to 0.9. Regarding the two existing methods, they could only achieve limited improvements compared with random channel access scheme, and the DQN-based scheme performs constantly better than the Q-learning method, since historical channel usage status is utilized. Besides the ideal results, it is obvious that the proposed scheme performs the best, which keeps a small performance gap to the ideal results. The gap becomes larger when the DC increases, since the whitespace is extremely limited for the cases that DC is 0.7 and 0.9. However, the proposed scheme still significantly outperforms the two existing methods at all DC values. When the DC is low, i.e., 0.1 and 0.3, we consider that the improvements mainly come from two aspects. One is the proposed compressed states representation scheme which results in learning with deeper historical channel usage status. Another is the proposed usage aware cost function design which reduces the cost value’s sparsity. When the DC is high, i.e., 0.5, 0.7 and 0.9, we can confirm that the proposed scheme has a further performance improvements compared to the existing methods. Aside from the two previously mentioned aspects, we consider the reason is that the additional “no access” action provides SU a new option to avoid interference by refraining from channel access.

Figure 6b–f show the results of $P_{s}-P_{i}$ at different DC by varying the complexities of the PU channel usage patterns. As expected, the ideal results and the performance of random channel access method keep constant regardless of the complexities. On the other hand, the performance of the two existing methods degrades as the PU’s channel usage complexity increases. This performance variation becomes large when the DC increases. For the high DC and high complexity cases, i.e., $\eta_{7}∼\eta_{9}$ when DC=0.7 and $\eta_{5}∼\eta_{9}$ when DC=0.9, their performance degrades to the same level as the random channel access method, which indicates that the learning has failed and the prediction results are not helpful. It is confirmed that the proposed scheme keeps a constant gap to the ideal results at all DCs for all the patterns with different complexities. The performance of the proposed scheme is not affected by the complexities. It performs well even in very challenging scenarios with high DC and high complexity.

**Figure 7:**
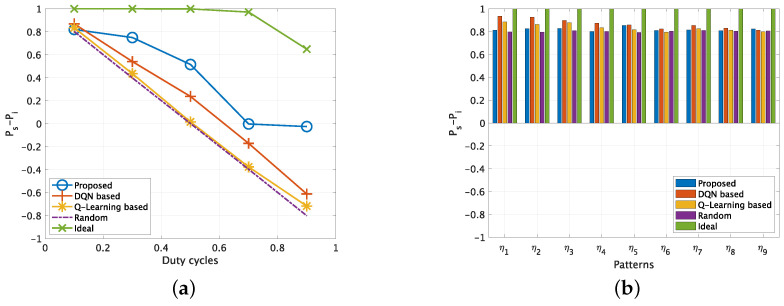

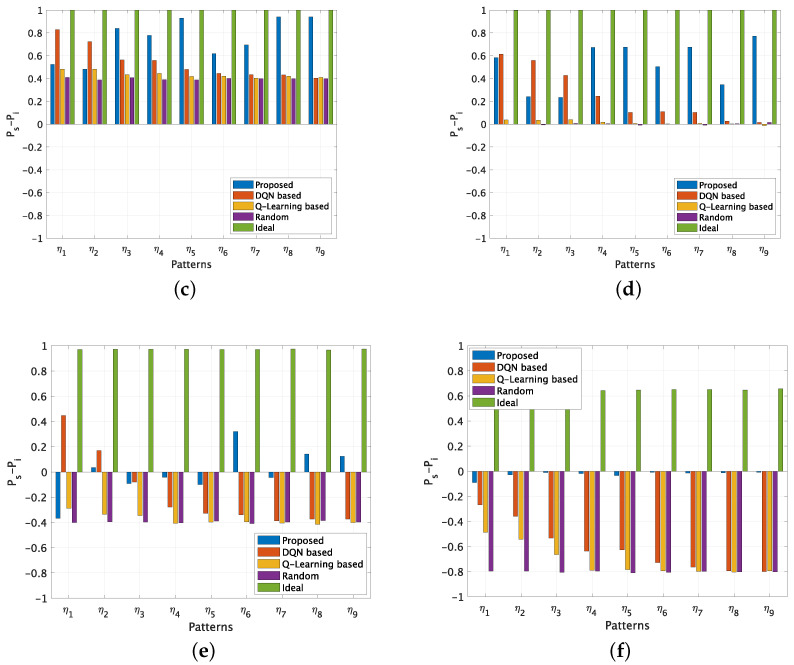
$P_{s}-P_{i}$ for uncorrelated PU channels (No. of PU=3). (**a**) Varying DCs. (**b**) Different patterns when DC = 0.1. (**c**) Different patterns when DC = 0.3. (**d**) Different patterns when DC = 0.5. (**e**) Different patterns when DC = 0.7. (**f**) Different patterns when DC = 0.9.

Finally, we present the performance evaluation results for a very challenging 10 uncorrelated PU channel scenario in Figure 7. Figure 7a shows the results of $P_{s}-P_{i}$ with varying DC from 0.1 to 0.9. Compared with the results for three PU channels scenario that is illustrated in Figure 6a, the performance of the proposed scheme degrades considerably, since the environment becomes extremely complicated and thus the state-action space increases exponentially. Specifically, the proposed scheme’s $P_{s}-P_{i}$ can only achieve approximately 0 when the DC is 0.7 or 0.9, which means that it is hard for the SU to predict the whitespace and it prefers no access action to avoid interference. The performance of the Q-learning based method is almost the same as the random access method, which means it cannot deal with such complicated scenario. Figure 7b–f show the results of $P_{s}-P_{i}$ at different DC by varying the complexities of the PU channel usage patterns. When the DC of PUs is 0.1, all the methods show the similar performance regardless of the complexities. For the cases that DCs of PU are 0.3 and 0.5, the proposed scheme can achieve comparatively satisfied performance compared with other schemes in all patters. The DQN-based method performs well in low complexity patterns, however, its performance degrades a lot when the complexity increases. Finally, when the DCs of PU are 0.7 and 0.9, accessing the channel without interference becomes extremely challenging. The learning results of the proposed method suggests the SU chooses no access action to avoid interference. Furthermore the Q-learning based and DQN-based methods have a great number of interference especially when the complexity is high.

## 6. Conclusions

In this paper, we proposed a novel deep reinforcement learning based usage aware spectrum access scheme for a typical multiple PUs and single SU cognitive radio network. Specifically, the proposed scheme consists of three key techniques which are compressed state representation, additional action option and status aware cost function design. By learning both the time and channel correlations of the PU channels, the proposed scheme is capable of reducing the spectrum underutilization and interference violations. We performed extensive simulations by considering both uncorrelated and correlated channel scenarios, and compared the performance of the proposed scheme with existing schemes and ideal results. The evaluation results showed that when PU = 3, the proposed scheme keeps a constant small performance gap between the ideal results, and significantly outperforms the existing methods especially at high PUs’ DC and complexity cases, e.g., a 3.28 times performance improvement over existing two schemes when DC = 0.9. However, regarding the results when PU = 10, the performance of the proposed scheme degrades significantly. In the worst case, i.e., when DC = 0.7, only a 1.22 times performance improvement over DQN-based method is obtained. For the future research, we plan to improve the performance of the proposed method in complicated scenarios by using deeper neural networks, and extend the proposed approach to combined channel access scenario and evaluate it by using real data traffic traces.

## Figures and Tables

**Table 1:** Entropy rates for different patterns at each DC.

DC	0.1	0.3	0.5	0.7	0.9
$\eta_{1}$	0.1261	0.3195	0.4689	0.3195	0.1261
$\eta_{2}$	0.2104	0.5119	0.4690	0.5119	0.2105
$\eta_{3}$	0.2777	0.6519	0.7219	0.6519	0.2777
$\eta_{4}$	0.3330	0.7539	0.7219	0.7539	0.3330
$\eta_{5}$	0.3788	0.8141	0.8813	0.8247	0.3788
$\eta_{6}$	0.4152	0.8247	0.8813	0.8141	0.4152
$\eta_{7}$	0.4152	0.8659	0.9710	0.8659	0.4431
$\eta_{8}$	0.4431	0.8669	0.9710	0.8669	0.4617
$\eta_{9}$	0.4617	0.8813	1.0000	0.8813	0.4690

**Table 2:** Main parameters for the proposed method.

Parameters	Value
Number of PUs	3, 10
Total time slots	110,000
Evaluation time slots	10,000
Mini-batch size	2500
Replay memory size	125,000
Exploration rate	1 → 0.001
Learning rate	0.00009
Discount factor	0.1
Number of hidden layers	1
Number of neurons	512
